# Essential Tremor as a “Waste Basket” Diagnosis: Diagnosing Essential Tremor Remains a Challenge

**DOI:** 10.3389/fneur.2020.00172

**Published:** 2020-03-25

**Authors:** Christian J. Amlang, Daniel Trujillo Diaz, Elan D. Louis

**Affiliations:** ^1^Department of Neurology, Yale School of Medicine, Yale University, New Haven, CT, United States; ^2^Department of Chronic Disease Epidemiology, Yale School of Public Health, Yale University, New Haven, CT, United States; ^3^Center for Neuroepidemiology and Clinical Neurological Research, Yale School of Medicine, Yale University, New Haven, CT, United States

**Keywords:** essential tremor, dystonia, Parkinson's disease, differential diagnosis, clinical

## Abstract

**Introduction:** The diagnosis of essential tremor (ET) remains a clinical one, and diagnostic errors are common. We aimed to (1) determine precisely how frequently ET diagnoses are misapplied (i.e., what percentage of patients who have been assigned an “ET” diagnosis actually have another movement disorder), (2) determine which other movement disorders are most often misclassified as “ET,” and (3) examine the clinical features that were most associated with diagnostic errors.

**Methods:** One hundred four consecutive patients were included who met the following criteria: (1) initial outpatient evaluation by one of the authors (EDL) between January 2015 and December 2019 and (2) pre-evaluation diagnosis of ET. Data on an extensive number of clinical features were extracted from the electronic medical record.

**Results:** Forty-seven (45.2%) patients received a post-evaluation diagnosis of ET, 29 (27.9%) of dystonia, and 28 (26.9%) of other diagnoses including Parkinson's disease (PD) [6 (5.8%)]. Factors associated with an alternative post-evaluation diagnosis other than ET were pre-evaluation diagnosis made by a non-neurologist, shorter tremor duration, irregular tremor, abnormal limb postures, among others.

**Discussion:** Diagnosing ET remains a challenge, with the diagnosis being over-applied and being used as a “waste basket.” More than one-half of the patients who were referred to our clinic with an intake diagnosis of ET were given an alternative post-evaluation diagnosis. While PD was reported to be the most frequently missed diagnosis in a past study, dystonia was most commonly missed in our study. Several clinical features can help to differentiate ET from other tremor disorders.

## Introduction

Essential tremor (ET) is one of the most common movement disorders, with a prevalence of 4.6% among persons aged 65 and older (1). The diagnosis remains a clinical one, and diagnostic errors are quite common, with frequent misclassification with respect to other movement disorders, especially Parkinson's disease (PD), dystonia, and enhanced physiologic tremor (2–4). In particular, earlier and mild stages of these diseases remain a diagnostic challenge, as clinical phenomenology is often subtler in these settings. While a variety of ancillary tests are used to support the diagnosis of PD, including 123I-FP-CIT (DaTSCAN) single-photon emission computerized tomography (5) and substantia nigra neuromelanin magnetic resonance imaging (6), there are still no specific biomarkers or imaging procedures to diagnose ET.

An earlier literature suggested that as many as 37–50% of “ET” cases have other neurological disorders, with PD being among the most common (2, 3). With the advent of greater societal awareness of PD and the widespread availability of DaTSCAN, however, it is quite probable that this is less often the case. Nevertheless, this topic has not been revisited in the past 15–20 years (2, 3), with no studies during the intervening period. Hence, there is a need for updated and renewed knowledge, with a fresh look at this topic. Our *a priori* hypothesis was that diagnostic errors with respect to ET would remain common, likely more than 25%, but that misdiagnosis with PD would be less of an issue than reported in earlier studies.

The aims of this study were to (1) determine how frequently “ET” diagnoses are misapplied (i.e., what percentage of patients who have been assigned an “ET” diagnosis actually have another movement disorder), (2) determine which other movement disorders were most often misclassified as “ET,” and (3) examine the clinical features that were most associated with diagnostic errors.

These results have a number of potential clinical implications. One of these is that misdiagnosis is the beginning of errors in treatment, and it is important for clinicians to be aware of and try to prevent such errors. Another clinical implication is that many of these illnesses in question (i.e., ET, dystonia, PD) have a familial component, so that arriving at the correct diagnosis has broader implications for offspring and other family members of patients, who themselves deserve to know, correctly, for which disease they may be at increased risk. Finally, issues related to the diagnosis of ET and its association with other movement disorders are increasingly at the forefront of the scientific dialogue on ET. We believe these data will contribute additional information, which will be of value in addressing this challenge.

## Methods

### Study Setting and Data Abstraction

The study setting was a clinical practice at an academic medical center, which served the local community and also served as a referral center. Through our university computerized clinical interface, we searched and identified all consecutive patients who fulfilled each of the following inclusion criteria: (1) initial outpatient evaluation by one of the authors (EDL), (2) pre-evaluation diagnosis of ET, (3) initial evaluation by EDL occurred between January 2015 (initiation of the author's practice at this medical center) and December 2019 (present). One of the authors (CJA) retrospectively extracted data from the patients' electronic medical records. The data included the post-evaluation diagnosis [assigned by EDL using diagnostic criteria from the International Parkinson and Movement Disorder Society (MDS) (MDS) consensus statement (7)].

We carefully pre-specified variables of interest and extracted these from the initial evaluation by EDL. See Table 1 for a detailed explanation of each clinical variable of interest.

**Table 1:** Explanation and hypothesis for the historical and clinical characteristics of the included patients.

**Criteria/characteristics**	**Explanation**	**Hypothesis**
Referral ET diagnosis made by a non-neurologist	Was the referral diagnosis of ET made by a neurologist, primary care physician, the patient or someone else?	Diagnoses made by a neurologist are more accurate than those made by a non-neurologist
Referral ET diagnosis made <1 year ago	Whether the diagnosis was established recently or more than 1 year ago	If the ET diagnosis was made only recently, it is more likely that the diagnosis may be inaccurate
Onset of tremor <5 years ago	Whether the tremor onset was more recent or more than 5 years ago	If the tremor began only recently, symptoms are more likely to be subtle and a misdiagnosis is more likely
ET medications tried	Whether any medications have been tried for ET	The previous trial of ET medications may increase the likelihood of an accurate diagnosis of ET
History: unilateral tremor	Whether the arm tremor started on one or on both sides of the body	Unilateral tremor is less likely to be ET
History: leg tremor	Whether leg tremor is present	Leg tremor is rare in ET (8, 9)
History: head tremor only	Presence of isolated head tremor (no limb tremor)	Isolated head tremor is not a feature of ET (10, 11)
History: head tremor started before the upper limb tremor	Whether the head tremor began before the upper limb tremor	If the head tremor started first, a diagnosis of dystonia (and therefore a misdiagnosis of ET) is more likely (11, 12)
History: handwriting impaired	Whether there is an impairment of the patient's handwriting	Impaired handwriting is non-specific and may be found in ET but also other disorders; however, absence of a handwriting impairment would be unusual in ET
Eastern European Jewish ancestry	Whether there is any Eastern European Jewish ancestry	Eastern European Jewish ancestry is associated with an increased risk of dystonia
Celtic ancestry	Whether there is any Celtic (Irish, Welsh, Scottish) ancestry	Celtic ancestry is associated with an increased risk of dystonia (13)
Examination: unilateral tremor	Whether the arm tremor is unilateral	Unilateral arm tremor is rare in ET (14)
Examination: leg tremor	Whether leg tremor is present	Leg tremor is rather rare in ET (8, 9)
Examination: head tremor only	Presence of isolated head tremor (no limb tremor)	Isolated head tremor is a rare feature of ET (10, 11)
Examination: tremor is irregular	Whether the tremor is non-rhythmic	ET is a rhythmic tremor (15)
Examination: rest tremor	Whether rest tremor is present	Rest tremor is generally found in more advanced ET cases (16)
Examination: head tremor improves while lying down	Whether the head tremor improves or stops when the patient lies down	The head tremor of ET often improves while the patient is supine (17)
Examination: head tremor has a directional quality	Whether the head tremor tends to be toward a specific side (right or left)	If the head tremor has a directional quality, the diagnosis of ET is likely inaccurate (18)
Examination: abnormal limb postures	If there are any abnormal body postures such as the spooning of the hand, torticollis, or abnormal posturing/flexion of the thumb, hand, or other body parts	The presence of abnormal postures is likely associated with a diagnosis of dystonia (and therefore a misdiagnosis of ET)
Examination: focal muscle hypertrophy	Whether there are any focal muscle hypertrophy/bulky muscles	The presence of focal muscle hypertrophy is likely associated with a diagnosis of dystonia (and therefore a misdiagnosis of ET)
Examination: one or more cardinal features of PD	Whether there are one or more cardinal features of PD	The presence of one or more cardinal features of PD is likely associated with a diagnosis of PD (and therefore a misdiagnosis of ET)
Examination: reduced arm swing	Whether there is reduced arm swing	If the arm swing is reduced on examination, the likely diagnosis is PD (and the ET diagnosis is not accurate)
Examination: slow rapid alternating movements	Whether there is bradykinesia during rapid alternating movements	The presence of bradykinesia suggests a diagnosis of PD (and the ET diagnosis is not accurate)
Examination: intention tremor	Whether intention tremor is present	Intention tremor is a common feature of ET; hence, the presence of an intention tremor is likely associated with an accurate diagnosis of ET (19)
Examination: single axis on spiral test	Whether there is a single identifiable axis present on the spiral test	The presence of a single axis on the spiral tests is likely associated with an accurate diagnosis of ET (20)
Examination: multiple axes on spiral test	Whether there are multiple axes on the spiral test	The presence of multiple axes on the spiral test is likely associated with a misdiagnosis of ET (21)
Examination: voice breaks or spasmodic voice	Whether the patient's voice has a spasmodic quality and/or there are voice breaks	The presence of voice breaks or a spasmodic voice is likely associated with a diagnosis of dystonia (and therefore a misdiagnosis of ET)

*ET, essential tremor; PD, Parkinson's disease*.

### Data Analysis

There were 104 patients. As a first analytic step, we pooled all 47 patients who had been assigned a post-evaluation diagnosis of “not-ET” by EDL into one group in order to evaluate which details of the history and the clinical examination were most associated with the non-ET diagnosis. Given that dystonia was the most commonly missed alternative diagnosis in our study population, we then conducted a separate analysis to evaluate which details of the history and the clinical examination were most associated with the dystonia diagnosis. Statistical analyses were performed using SPSS Version 24.0 (Tables 1–**4**). Student *t*-tests were used to analyze continuous variables and χ^2^ as well as Fisher exact tests were used for comparison of categorical variables. Non-parametric tests (e.g., Mann–Whitney test) were used when appropriate. Binary logistic regression analyses were performed in order to assess the relationship between each clinical characteristic and diagnosis (**Tables 3**, **4**). Each binary logistic regression analysis yielded an odds ratio (OR) and 95% confidence interval (CI) (**Tables 3**, **4**). We also repeated each of these logistic regression models, including gender as a covariate, but this did not appreciably change the results; hence, those data are not reported.

## Results

### Demographic Characteristics

We identified 104 consecutive patients. Their demographic characteristics are shown (Table 2).

**Table 2:** Demographic data on 104 patients.

**Data**	**Post-evaluation diagnosis = ET**	**Post-evaluation diagnosis = not ET**	**Post-evaluation diagnosis = dystonia**	**Post-evaluation diagnosis = other diagnoses aside from dystonia**
*n*	47 (45.2%)	57 (54.8%)	29 (27.9%)	28 (26.9%)
Age in years (mean values)	68.5 ± 16.0	64.5 ± 13.2	63.9 ± 12.2	65.0 ± 14.3
Age in years (median values)	72.0	67.0	66.0	69.0
Sex				
Female	19 (40.4%)	30 (56.8%)	19 (65.5%)	11 (39.3%)
Male	28 (59.5%)	27 (47.4%)	10 (34.5%)	17 (60.7%)

### Diagnostic Validity

Of the 104 patients, 47 (45.2%) were diagnosed with ET, 29 (27.9%) with dystonia, and 28 (26.9%) with other diagnoses including PD (6 or 5.8%), ET-PD (4 or 3.9%), ET and dystonia (3 or 2.9%), functional tremor (2 or 1.9%), primary writing tremor (2 or 1.9%), parkinsonian syndrome (1 or 1.0%), multiple system atrophy (1 or 1.0%), myoclonus dystonia (1 or 1.0%), enhanced physiologic tremor (1 or 1.0%), and uncertain diagnoses (7 or 6.7%).

### Clinical Characteristics of 47 Patients With a Post-evaluation Diagnosis of Essential Tremor vs. 57 Patients With All Other Post-evaluation Diagnoses

Patients who received a post-evaluation diagnosis of “not ET” differed in a number of respects from patients who received a post-evaluation diagnoses of ET (Tables 2, 3). Patients who received a post-evaluation diagnosis of ET were statistically similar regarding their sex distribution (*p* = 0.21) and were of similar age compared to their counterparts who received a post-evaluation diagnosis of “not ET” (*p* = 0.064). Patients who received a post-evaluation diagnosis of “not ET” were 3.8 times more likely to have previously been diagnosed by a non-neurologist than were patients who received a post-evaluation diagnosis of ET (Table 3). Patients with a post-evaluation diagnosis of “not ET” were 4.6 times more likely to have had tremor of short duration (i.e., <5 years) than were patients who received a post-evaluation diagnoses of ET (Table 3).

**Table 3:** Additional clinical characteristics of 47 patients with a post-evaluation diagnosis of ET versus 57 patients with all other post-evaluation diagnoses.

**Characteristics**	Post-evaluation diagnosis = ET (*n* = 47)^*^	Post-evaluation diagnosis = not ET (*n* = 57)^*^	OR ^**^ (95% confidence interval)	***p*-value**
Referral ET diagnosis made by a non-neurologist	7 (16.3%)	23 (42.6%)	3.8 (1.4–10.1)	0.007
Referral ET diagnosis made <1 year ago	7 (35.0%)	19 (55.9%)	2.4 (0.75–7.4)	0.14
Onset of tremor <5 years ago	6 (12.8%)	23 (40.3%)	4.6 (1.7–12.7)	0.003
ET medications tried	34 (72.3%)	34 (59.6%)	1.8 (0.8–4.1)	0.18
History: unilateral tremor	1 (2.1%)	14 (24.6%)	15.0 (1.9–118.8)	0.010
History: leg tremor	1 (2.1%)	7 (12.3%)	6.4 (0.8–54.4)	0.09
History: head tremor only	0 (0.0%)	5 (8.8%)	10.0 (0.5–184.8)	0.12
History: head tremor started before upper limb tremor	1 (2.1%)	5 (8.8%)	3.9 (0.4–41.3)	0.26
History: handwriting impaired	21 (44.7%)	18 (31.6%)	1.8 (0.8–3.9)	0.17
Eastern European Jewish ancestry	3 (6.4%)	10 (17.5%)	3.1 (0.8–12.1)	0.10
Celtic ancestry	10 (21.3%)	10 (17.5%)	1.3 (0.5–3.4)	0.63
Examination: unilateral tremor	0 (0.0%)	7 (13.0%)	14.7 (0.8–264.5)	0.07
Examination: leg tremor	1 (2.1%)	4 (7.0%)	3.5 (0.4–32.2)	0.27
Examination: head tremor only	0 (0.0%)	3 (5.3%)	6.1 (0.3–121.2)	0.24
Examination: tremor is irregular	0 (0.0%)	19 (33.3%)	48.1 (2.8–822.9)	0.008
Examination: rest tremor	7 (14.9%)	16 (28.1%)	2.2 (0.8–6.0)	0.11
Examination: head tremor improves while lying down	8 (17.0%)	9 (15.8%)	1.2 (0.4–4.3)	0.73
Examination: head tremor has a directional quality	3 (6.4%)	12 (21.1%)	3.9 (1.0–14.8)	0.045
Examination: abnormal limb postures	13 (27.7%)	35 (61.4%)	4.1 (1.8–9.6)	0.001
Examination: focal muscle hypertrophy	1 (2.1%)	8 (14.0%)	7.5 (0.9–62.4)	0.062
Examination: one or more cardinal features of PD	3 (6.4%)	15 (26.3%)	5.2 (1.4–19.4)	0.013
Examination: reduced arm swing	13 (27.7%)	18 (31.6%)	1.2 (0.5–2.8)	0.66
Examination: slow rapid alternating movements	8 (17.0%)	17 (30.0%)	2.1 (0.8–5.4)	0.13
Examination: intention tremor	31 (66.0%)	24 (42.1%)	0.4 (0.2–0.8)	0.016
Examination: single axis on the spiral test	33 (82.5%)	12 (27.3%)	0.1 (0.03–0.2)	0.001
Examination: multiple axes on the spiral test	2 (5.0%)	13 (29.5%)	8.0 (1.7–38.0)	0.009
Examination: voice breaks or spasmodic voice	0 (0.0%)	6 (10.5%)	21.0 (1.0–438.3)	0.0495

*ET, essential tremor; OR, odds ratio*.

* *In a small number of patients, data were missing. Percentages and ORs reflect the n with complete data*.

** *OR reflects non-ET/ET*.

By history, patients who were assigned a post-evaluation of “not ET” were more likely to have unilateral tremor (OR = 15.0). The presence of a leg tremor (OR = 6.4) or isolated head tremor (OR = 10.0) also seemed to be more common among “non-ET” patients; but despite the large ORs, in each of these comparisons, *p*-values were not statistically significant (*p* = 0.09 and *p* = 0.12) (Table 3).

On examination, a unilateral tremor seemed to be more common in patients who received a post-evaluation diagnosis of “not ET”; however, the difference did not reach a statistically significant level (OR = 14.7, *p* = 0.07) (Table 3). If the observed head tremor was of an irregular rhythmicity, it was far more likely not be classified as ET after evaluation in our clinic (OR = 48.1) (Table 3). The presence of typical dystonic features such as abnormal limb postures (OR = 4.1) was associated with post-evaluation diagnoses of “not ET” (Table 3). PD was the second most common alternative diagnosis [six cases (10.5%)], and it was 5.2 times more likely that the post-evaluation diagnosis was not ET if at least one of the cardinal features of PD was present on examination (Table 3). An intention tremor on examination was less likely to be found in patients who received a post-evaluation diagnosis of “not ET” (OR = 0.4), as was a single axis on the spiral test (OR = 0.1), whereas multiple axes on the spiral test were significantly associated with a post-evaluation diagnosis of “not ET” (OR = 8.0) (Table 3).

### Clinical Characteristics of 47 Patients With a Post-evaluation Diagnosis of Essential Tremor vs. 29 Patients With a Post-evaluation Diagnosis of Dystonia

When compared with patients with a post-evaluation diagnosis of dystonia, a larger proportion of patients with a post-evaluation diagnosis of ET were male (*p* = 0.026). The two groups were also different in age (*p* = 0.046) (Table 2).

Patients who had been previously diagnosed by a non-neurologist seemed to be more likely to have a post-evaluation diagnosis of dystonia (OR = 2.7) even though this did not reach statistical significance (*p* = 0.08) (Table 4). By history, it was significantly more likely that a patient would be eventually diagnosed with dystonia if he or she reported the presence of an isolated head tremor (OR = 21.3) (Table 4). With regard to family history and ancestry, patients with a post-evaluation diagnosis of dystonia were more likely to have been of Eastern European Jewish ancestry than were those with a post-evaluation diagnosis of ET (OR = 5.6) (Table 4).

**Table 4:** Additional clinical characteristics of 47 patients with a post-evaluation diagnosis of ET vs. 29 patients with a post-evaluation diagnosis of dystonia.

**Characteristics**	Post-evaluation diagnosis = ET (*n* = 47)^*^	Post-evaluation diagnosis = dystonia (*n* = 29)^*^	OR ^**^ (95% confidence interval)	***p*-value**
Referral ET diagnosis made by a non- neurologist	7 (16.3%)	10 (34.5%)	2.7 (0.9–8.2)	0.08
Referral diagnosis made <1 year ago	7 (35.0%)	9 (50.0%)	1.9 (0.5–6.8)	0.35
Onset of tremor <5 years ago	6 (12.8%)	7 (24.1%)	2.2 (0.7–7.3)	0.21
ET medications tried	34 (72.3%)	17 (58.6%)	0.5 (0.2–1.4)	0.22
History: unilateral tremor	1 (2.1%)	2 (6.9%)	3.4 (0.3–39.4)	0.33
History: leg tremor	1 (2.1%)	1 (4.5%)	1.6 (0.1–27.3)	0.73
History: head tremor only	0 (0.0%)	5 (17.2%)	21.3 (1.1–401.7)	0.041
History: head tremor started before the upper limb tremor	1 (2.1%)	3 (10.3%)	7.0 (0.5–97.8)	0.15
History: handwriting impaired	21 (44.7%)	9 (31.0%)	0.6 (0.2–1.5)	0.24
Eastern European Jewish ancestry	3 (6.4%)	8 (27.6%)	5.6 (1.3–23.2)	0.018
Celtic ancestry	10 (21.3%)	9 (31.0%)	1.7 (0.6–4.8)	0.34
Examination: unilateral tremor	0 (0.0%)	0 (0.0%)		
Examination: leg tremor	1 (2.1%)	0 (0.0%)	0.5 (0.0–13.3)	0.70
Examination: head tremor only	0 (0.0%)	3 (10.3%)	12.5 (0.6–252.3)	0.01
Examination: tremor is irregular	0 (0.0%)	14 (48.3%)	88.9 (5.0–1,578.4)	0.002
Examination: rest tremor	7 (14.9%)	3 (10.3%)	0.7 (0.2–2.8)	0.57
Examination: head tremor improves while lying down	8 (17.0%)	6 (20.7%)	0.8 (0.2–3.0)	0.68
Examination: head tremor has a directional quality	3 (6.4%)	9 (31.0%)	6.6 (1.6–27.0)	0.009
Examination: abnormal limb postures	13 (27.7%)	24 (82.8%)	12.6 (4.0–40.0)	0.001
Examination: focal muscle hypertrophy	1 (2.1%)	3 (10.3%)	5.3 (0.5–53.7)	0.16
Examination: one or more cardinal features of PD	3 (6.4%)	2 (6.9%)	1.1 (0.2–6.9)	0.93
Examination: reduced arm swing	13 (27.7%)	6 (20.7%)	0.7 (0.2–2.1)	0.50
Examination: slow rapid alternating movements	8 (17.0%)	3 (10.3%)	0.6 (0.1–2.3)	0.43
Examination: intention tremor	31 (66.0%)	13 (44.8%)	0.4 (0.2–1.1)	0.073
Examination: single axis on the spiral test	33 (82.5%)	4 (16.6%)	0.04 (0.01–0.2)	0.001
Examination: multiple axes on the spiral test	2 (5.0%)	11 (45.8%)	16.1 (3.1–82.3)	0.001
Examination: voice breaks or spasmodic voice	0 (0.0%)	6 (20.7%)	54.6 (2.2–1,326.3)	0.014

*ET, essential tremor; OR, odds ratio*.

* *In a small number of patients, data were missing. Percentages and ORs reflect the n with complete data*.

** *OR reflects dystonia/ET*.

On examination, an irregular rhythmicity was strongly associated with a post-evaluation diagnosis of dystonia (OR = 88.9, *p* = 0.002) as was the presence of an isolated head tremor (OR = 12.5, *p* = 0.01) (Table 4). When present, it did not make a difference whether the head tremor improved or resolved when lying down; however, when the head tremor had a directional quality, it was 6.6 times more likely to be diagnosed as a dystonic tremor (Table 4). Similarly, the presence of abnormal upper limb postures such as spooning of the hand or hyperflexion of the thumb was strongly associated with a post-evaluation diagnosis of dystonia (OR = 12.6, *p* = 0.001) (Table 4).

The majority of patients did not have a voice tremor [81/104 (77.9%)]; however, when a voice tremor was present and had a spasmodic quality or if there were voice breaks, the patient was 54.6 times more likely to have a post-evaluation diagnosis of dystonia (Table 4). Patients with a post-evaluation diagnosis of dystonia were 16.1 times more likely to have multiple axes on their spiral test (Table 4).

## Discussion

Our study indicates that diagnosing ET remains a challenge. Indeed, the diagnosis is over-applied and used as somewhat of a “waste basket” for a variety of tremor disorders. More than one-half of the patients who were referred to our movement disorders clinic with an intake diagnosis of ET were given an alternative diagnosis at the end of their encounter. Dystonia was the most commonly previously missed diagnosis followed by PD. Interestingly, a previous study with a similar methodology (3) reported that PD was the most commonly missed diagnosis—in that study, 15% of the cases had pure PD and 7% had PD with ET. It is possible that nowadays PD is more accurately diagnosed as a result of greater societal awareness and new ancillary tests, as noted above. Nevertheless, in another prior study with a different methodological approach (2), it was already estimated that dystonia was the most frequent differential diagnosis of ET, such as in our current study.

Our data also illustrate that an ET diagnosis is more valid when assigned by a neurologist and when the tremor has been present for a longer period of time (more than 5 years). The latter point seems to confirm the diagnostic challenges in tremor patients when the symptom onset is recent and accompanying clinical features, which might help to ascertain the correct diagnosis, are subtle or not yet present.

Patients with ET can during the course of their illness develop signs of dystonia and parkinsonism, and some may even develop a secondary PD (22, 23). This is generally a later phenomenon. We report a discrepancy between intake and post-evaluation diagnosis of ET. It is possible that some of the intake diagnoses were assigned many years prior to seeing us, and that dystonic features and parkinsonism postdated those assessments. Indeed, in more than one-half of our patients with discrepant diagnoses, the latency from initial diagnosis to our evaluation was 20 or more years. This could explain some of the discrepancy we see between intake and post-evaluation diagnoses. However, a sizable proportion of our patients had clear dystonia on examination, primary writing tremor, functional tremor, or myoclonus, and this was their post-evaluation diagnosis; we believe that the intake diagnosis in those was simply incorrect due to a failure on the part of the clinician to recognize their clinical features.

Both dystonia and ET are purely clinical diagnoses as there is no specific ancillary test. While the spiral test can help distinguish between the two conditions (21), this test has only modest diagnostic validity. Certain clinical features of the tremor such as its amplitude or the presence of spooning and other abnormal postures (20, 24) can also help in the differentiation between ET and dystonia. One of the main reasons why dystonia is frequently misdiagnosed as ET may be that ET is such a common disease [with an estimated 4% prevalence of ET among individuals older than 60 years (1) and an estimated prevalence of dystonia of approximately 0.4% (25)]. According to our data, several historical and clinical details were more likely to be associated with dystonia and might be useful to establish the correct diagnosis. Features that indicated a likely diagnosis of dystonia were isolated head tremor, Eastern European Jewish ancestry [see (26)], lack of rhythmicity, directionality of the tremor, abnormal postures [see (24, 27)], voice breaks or a spasmodic voice, and multiple axes on the spiral test compared to one single axis in ET patients (20, 21). Of note, while the presence of an intention tremor in patients with true ET helped to differentiate them from patients with any non-ET diagnosis, this was not the case when comparing ET to dystonia patients, which is in line with recent evidence that implies the existence of a cerebellar dysfunction in dystonia (28).

There are certain limitations to our study. First, the patients were evaluated at a tertiary referral center, which might indicate that their cases were more complex and therefore the probability of a misdiagnosis higher than for patients with a diagnosis of ET for whom it was thought to be unnecessary to refer them to a higher level of care. However, the study setting was a clinical practice at an academic medical center, which in addition to being a tertiary referral center also served the local community, so this critical comment is not totally valid. Second, all of our patients were evaluated by solely one provider (EDL), so it is difficult to generalize these results to all settings and all practitioners. Nonetheless, this study design resulted in a high degree of final diagnostic validity as the provider has a long-standing interest and deep knowledge of ET and its diagnostic nuances. Hence, this design feature was a relative strength of the study rather than a limitation. Third, the number of patients we included in our study was small, which is why some of the differences, though accompanied by high ORs, did not reach statistical significance. Despite this, in Tables 3, 4, 20 comparisons were statistically significant. This represents a full 37% of all 54 comparisons, and another six (11%) had *p*-values that were between 0.05 and 0.10. Larger samples would even further facilitate statistical testing and help in evaluating the role of chance in comparisons that were bordering on significant.

Nonetheless, our study provides important evidence that ET is still frequently overdiagnosed and that the correct diagnosis of alternative movement disorders is oftentimes missed, particularly when the initial ET diagnosis was established by someone who is not a neurologist and when the tremor onset is recent. This observation is not of purely academic interest given that the treatment for ET, dystonia, PD, or other tremor disorders is very different. The importance of the aforementioned point becomes evident when taking into consideration that there was no difference between our patients with a true diagnosis of ET or a non-ET diagnosis regarding the question of whether they had been tried on ET medications before being evaluated in our clinic. This means not only that did they not receive the appropriate therapy for their condition but also that they were started on a treatment that could have induced side effects without providing a potential symptomatic benefit.

Ultimately, raising the awareness of specific clinical features and ancillary tests that can be useful in establishing the correct diagnosis of a tremor condition would be an important step to increase the accuracy of ET and non-ET diagnoses and to shorten the time period between tremor onset and initiation of adequate therapy if necessary.

## Data Availability Statement

The datasets generated for this study are available on request to the corresponding author.

## Ethics Statement

The studies involving human participants were reviewed and approved by Yale IRBs– Yale University Institutional Review Boards. Written informed consent for participation was not required for this study in accordance with the national legislation and the institutional requirements.

## Author Contributions

All authors listed have made a substantial, direct and intellectual contribution to the work, and approved it for publication.

### Conflict of Interest

The authors declare that the research was conducted in the absence of any commercial or financial relationships that could be construed as a potential conflict of interest.
